# Hedgehog pathway in sarcoma: from preclinical mechanism to clinical application

**DOI:** 10.1007/s00432-023-05441-3

**Published:** 2023-10-10

**Authors:** Natalia Banaszek, Dominika Kurpiewska, Katarzyna Kozak, Piotr Rutkowski, Paweł Sobczuk

**Affiliations:** 1 Department of Soft Tissue/Bone Sarcoma and Melanoma, Maria Skłodowska-Curie National Research Institute of Oncology in Warsaw, Warsaw, Poland; 2 Faculty of Medicine, Medical University of Warsaw, Warsaw, Poland

**Keywords:** Sarcoma, Soft-tissue sarcoma, Bone sarcoma, Hedgehog pathway, Gli

## Abstract

Sarcomas are a diverse group of malignant neoplasms of mesenchymal origin. They develop rarely, but due to poor prognosis, they are a challenging and significant clinical problem. Currently, available therapeutic options have very limited activity. A better understating of sarcomas’ pathogenesis may help develop more effective therapies in the future. The Sonic hedgehog (Shh) signaling pathway is involved in both embryonic development and mature tissue repair and carcinogenesis. Shh pathway inhibitors are presently used in the treatment of basal cell carcinoma. Its increased activity has been demonstrated in many sarcomas, including osteosarcoma, Ewing sarcoma, chondrosarcoma, rhabdomyosarcoma, leiomyosarcoma, and malignant rhabdoid tumor. In vitro studies have demonstrated the effectiveness of inhibitors of the Hedgehog pathway in inhibiting proliferation in those sarcomas in which the components of the pathway are overexpressed. These results were confirmed by in vivo studies, which additionally proved the influence of Shh pathway inhibitors on limiting the metastatic potential of sarcoma cells. However, until now, the efficacy of sarcomas treatment with Shh pathway inhibitors has not been established in clinical trials. The reason for that may be the non-canonical activation of the pathway or interactions with other signaling pathways, such as Wnt or Notch. In this review, we present the Shh signaling pathway's role in the pathogenesis of sarcomas, including both canonical and non-canonical signaling. We also propose how this knowledge could be potentially translated into clinics.

## Introduction

Signaling pathways, crucial in the physiological functions of cells and tissues, may, through dysregulation and ensuing dysfunctions, be significant factors in the process of tumorigenesis (Park et al. 2020). The Hedgehog (Hh) signaling pathway plays an important role in embryogenesis and in the upkeep of mature tissues and stem cells (Katoh and Katoh 2008). Mutations leading to dysregulation of the Hh pathway are consistently observed in the basal cell carcinoma (BCC) and medulloblastoma and sporadically in other cancers (Carpenter and Ray 2019). Currently, treatment with specific Shh pathway inhibitors has been approved in BCC by both European Medical Agency (EMA) and Food and Drug Administration (FDA) (Brancaccio et al. 2020), but new studies are being carried out in the hope of expanding these indications (Carballo et al. 2018).

Sarcomas develop from transformed mesenchymal cells and are usually divided into sarcomas arising from the soft tissues and the bones. They are further segregated into various subtypes, making them a very diverse group of tumors (Mehren et al. 2020; Gronchi et al. 2021; W.C.o.T.E. Board 2020). Though sarcomas are rare, accounting for just 1% of all adult malignant tumors, they are characterized by poor prognosis and unsatisfactory treatment options, which makes them a significant clinical challenge (Mastoraki et al. 2020).

In this article, we summarize the current data regarding the role played by the Shh pathway in the pathogenesis of sarcomas. Our goal is to emphasize that connection and start a discussion about the potential value of Shh targeted therapy in the treatment of sarcomas.

## Sonic Hedgehog pathway activation and regulation

The Hedgehog (Hh) pathway is a ligand-dependent signaling pathway. In vertebrates, three Hh ligands have been described: Desert hedgehog (Dhh), Indian hedgehog (Ihh), and Sonic hedgehog (Shh), the last one being the primary ligand in humans (Lézot et al. 2020). Furthermore, the Hh signaling pathway consists of two 12-pass transmembrane receptors Ptch1 and Ptch2, Smoothened (Smo) receptor, and three transcription factors Gli1, Gli2, and Gli3 (Yao et al. 2018).

**Fig. 1:**
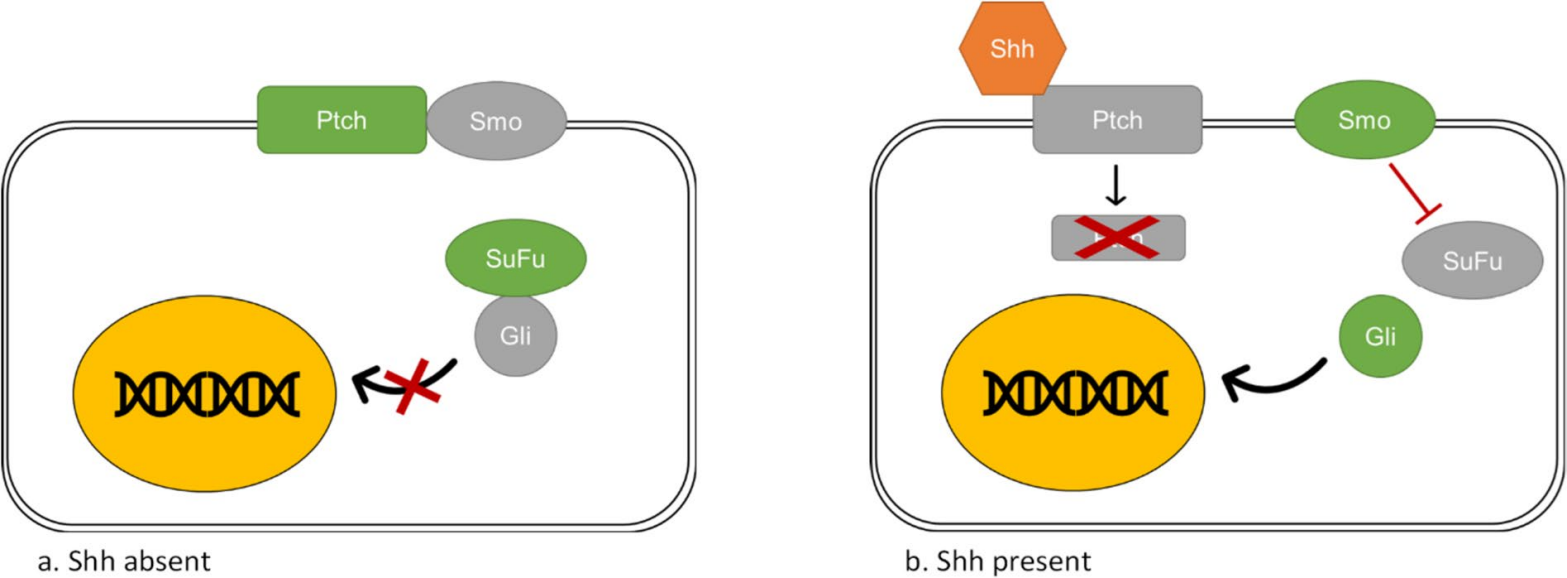
Shh pathway activation. The Shh pathway consists of the Sonic hedgehog ligand (Shh), Patched receptor (Ptch), Smoothened receptor (Smo), Suppressor of fused (SuFu) and transcription factors (Gli). The illustration shows the canonical activation of the Shh signaling pathway. **a** In the absence of the Shh ligand, Ptch binds to a constitutively active Smo, suppressing its activity. In the absence of activated Smo, SuFu forms SuFu–Gli complexes and restrains Gli activity. **b** In the presence of the Shh ligand, its binding with Ptch results in endocytic degradation of Ptch in the lysosome. Activated Smo induces the dissociation of SuFu–Gli complexes and translocation of Gli proteins into the nucleus

There are two pathways which activate the Shh cascade—a canonical and a non-canonical one (Lézot et al. 2020). Interaction between the Shh and the Ptch receptor is the base of the canonical cascade (Marigo et al. 1996). When the Hh ligand is absent, the Ptch receptor binds to a constitutively active Smo receptor, suppressing the Smo activity (Fig. 1) (Liu et al. 2017). The suppressor of fused (SuFu) is a negative regulator of the Shh pathway (Liao et al. 2020). In the absence of activated Smo, SuFu forms SuFu–Gli complexes and sequesters Gli proteins in the cytoplasm, restraining their activity (Zhang et al. 2017). However, recent data indicate that SuFu may also further increase Gli2 expression in cells with an already high Gli2 expression (Yin et al. 2019). The presence of the Hh ligand leads to the binding of the Hh ligand to the Ptch receptor, which results in endocytic degradation of Ptch in the lysosome (Incardona et al. 2000). Activated Smo induces the dissociation of SuFu–Gli complexes and translocation of Gli proteins into the nucleus, where it can regulate the expression of various genes (Ruel and Thérond 2009).

**Fig. 2:**
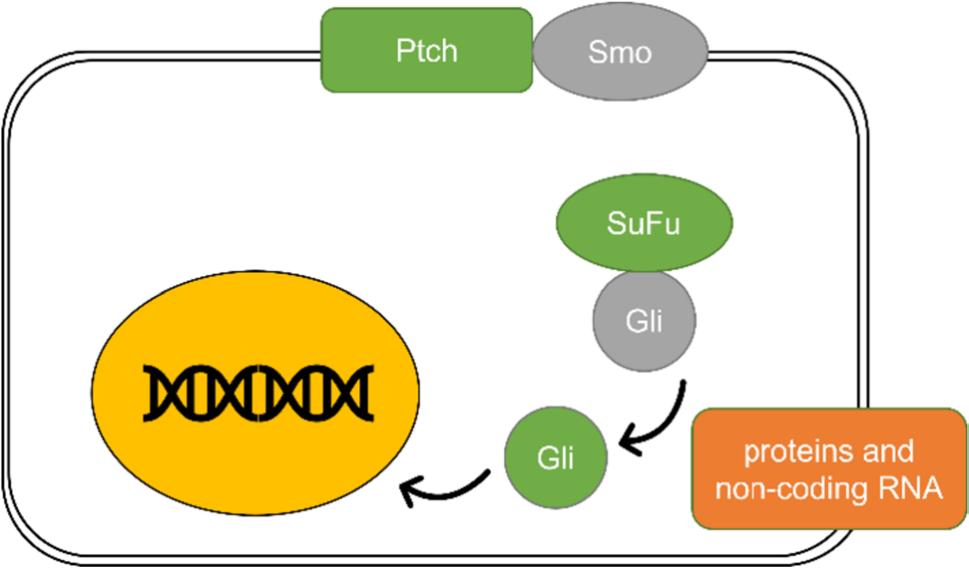
Non-canonical Shh pathway activation. The illustration shows an alternative way of Shh signaling pathway activation. The non-canonical pathway activation happens independently of Smo and directly through the activation of Gli by various proteins and non-coding RNA, some of which are described in more detail in the text

The non-canonical cascade refers to the activation of Gli transcription factors independently of Smo (Pietrobono et al. 2019). This can be caused by various signaling molecules and pathways, which can work separately or simultaneously (Pietrobono et al. 2019). Examples of these mechanisms will be discussed in more detail later. Non-canonical activation is involved in carcinogenesis connected with elevated Gli activity (Brechbiel et al. 2014) (see Fig. 2).

Various proteins and non-coding RNA regulate the Hh signaling pathway: microRNA (miRNA) and long non-coding RNA (lncRNA) (Yao et al. 2018). Notably, the regulators acting beyond Smo may participate in the non-canonical activation of the Shh pathway.

Kinases, transcription factors, glycoproteins, and pro- and anti-apoptotic factors have been connected to the activation or inhibition of the Hh pathway (Yao et al. 2018). Nek2A, an NIMA (never in mitosis gene a)-related kinase 2A, inhibits the Hh pathway and the transcriptional activity of Gli2 by stabilizing SuFu (Zhou et al. 2017). Rab23, a GTPase of the Rab family, also negatively regulates the Hh pathway through interaction with SuFu (Chi et al. 2012). Moreover, the methylation of Gli3, catalyzed by Set7, increases Gli3 stability and DNA-binding activity, which promotes the Shh pathway activation (Fu et al. 2016). Increased levels of Galectin-1 (Gal-1) activate the Hh signaling by increasing the transcription of Gli1 via a Smo-independent pathway (Chong et al. 2016). Another study demonstrated that silencing the B4GALT1 gene, which encodes Beta-1,4-galactosyl-transferase 1, in K562/ADR cells results in the inhibition of the Hh pathway (Zhou et al. 2012).

Proteins that interact directly with Smo also play a role in regulating the Hh pathway. RACK1, a receptor of activated kinase 1, initiates Gli1 transcription through interaction with activated Smo (Shi et al. 2012), while a GTPase Arl13b promotes Hh signaling by stabilizing Smo (Shao et al. 2017).

Gli1, Gli2, and Gli3 are also directly regulated by various proteins. Overexpression of NUSAP1 leads to upregulation of the Hh pathway’s target gene through induction of Gli1 translocation into the nucleus (Wu et al. 2017). Mastermind-like 1 (Maml1) binds to the Gli proteins and acts as a transcriptional coactivator, reinforcing the activation of Shh pathway target genes (Quaranta et al. 2017). Overexpression of beta1 integrin (ITGB1) results in the upregulation of Shh and Gli1 levels and the downregulation of SuFu, leading to the activation of Hh signaling (Song et al. 2015). FOXC1, a transcription factor, activates the Smo-independent Hh pathway by direct interaction with Gli2, increasing its DNA-binding and transcription-activating capacity (Han et al. 2015). Overexpression of Sloan–Kettering viral oncogene homolog (Ski), a protein which can function both as an oncoprotein and a tumor suppressor gene, leads to increased expression of Shh pathway components, such as Shh, Ptch-1, Smo, Gli1, and Gli2 (Song et al. 2016). Increased activity of mTORc2, one of the complexes formed by mTOR kinase (Fu and Hall 2020), has been shown to enhance the expression of Hh components (Gli1, Gli2, and Ptch1) and the target genes of the pathway (Cyclin D1, Cyclin D2, Cyclin E, Snail, Slug, and VEGF). Moreover, mTORc2 promotes stability and nuclear translocation of Gli2 (Maiti et al. 2017).

The hedgehog signaling pathway is crucial in embryonic processes: it controls cells differentiation, tissue polarity, and proliferation (Varjosalo and Taipale 2008). Timing aberrations of hedgehog signaling can generate embryological malformations (Ericson et al. 1996). To some degree, the Hh pathway remains active in mature organisms and participates in processes such as stem cell maintenance and tissue repair (Roma et al. 2012). Genes regulated by the Hh signaling pathway are crucial in cell proliferation and survival, cell cycle, cell invasion, and stem cell formation (Yao et al. 2018). However, in most tissues, the pathway remains inactive and is only activated when necessary, for example, in the regeneration of damaged tissues (Skoda et al. 2018).

The Hh pathway plays an essential role in osteogenesis, regulating endochondral and intramembranous ossification. It also promotes bone resorption through indirect activation of osteoclasts, making it a crucial factor in bone homeostasis and remodeling (Yang et al. 2015).

## The role of Shh pathway in the pathogenesis of selected subtypes of sarcomas

### Osteosarcoma 

Osteosarcoma is a malignant bone tumor that produces osteoid and immature bone and comprises mesenchyme-derived cells (W.C.o.T.E. Board 2020; Biazzo and Paolis 2016). It is a high-grade sarcoma and appears mostly among children and young adults (Lo et al. 2014a). Osteosarcoma has a high metastatic potential; approximately one-fourth of patients with osteosarcoma have metastases at presentation (Tsukamoto et al. 2020). The clinical outcome for patients with lung metastases remains poor, since they are hard to control (Yao et al. 2018) and are resistant to standard chemotherapy (Saitoh et al. 2016).

Previous studies have shown that both the canonical and non-canonical Hh pathways may be involved in osteosarcoma tumorigenesis (Lo et al. 2014b). Osteosarcoma patients with higher levels of Gli1 are more likely to respond better to chemotherapy (Lézot et al. 2020; Lo et al. 2014a). Other evidence suggests a correlation between overexpression of Gli-2 and poor clinical outcomes (Yang et al. 2013). Gene expression analyses by real-time PCR revealed overexpression of Shh, Ihh, Ptch1, Smo, and Gli2 in osteosarcoma cell lines. In contrast, examination of osteosarcoma biopsy specimens showed overexpression of Smo, Ptch1, and Gli compared to normal bone tissue cells (Hirotsu et al. 2010) (Yang et al. 2013). Another group confirmed higher expression of Gli1 and Gli2 in canine osteosarcoma cell lines compared to normal canine osteoblasts. Moreover, there was a correlation between the Gli1 or Gli2 expression level and the expression of Ptch1 and PAX6 (Shahi et al. 2014).

Data report that Gli2 significantly promotes the proliferation, migration, and invasion of mesenchymal stem cells and osteosarcoma cells (Nagao-Kitamoto et al. 2015a). This has been confirmed by knockdown of Gli2, which promoted the arrest of osteosarcoma cells in the G1 phase of cell cycle and inhibited osteosarcoma growth, demonstrated in murine xenograft models (Nagao et al. 2011). One of the target genes of Gli2 is the *RPS3* gene encoding the ribosomal protein S3, which is a component of the eukaryotic 40S ribosomal subunit and is involved in various processes, such as apoptosis, immune response, DNA repair, transcriptional regulation, and transformation (Yao et al. 2018; Gao and Hardwidge 2011). Overexpression of RPS3 increases the migration and invasion of osteosarcoma cells, playing a role in metastases formation (Nagao-Kitamoto et al. 2015a).

Exosomes, small vesicles secreted by various cells, contain proteins, lipids, and nucleic acids such as mRNA or miRNA. Exosomes acquired from mesenchymal stem cells derived from human bone marrow (hBMSC) have been shown to promote osteosarcoma cell growth by activating the Hh pathway. Hsp70 and CD63 expression has been detected in these exosomes (Qi et al. 2017).

The interaction of the Hh pathway with other signaling pathways can also be an essential factor in osteosarcoma progression, including metastasis formation (Yao et al. 2018). Some evidence suggests that aberrant Hh signaling leads to overexpression of the Yes-associated protein 1 (Yap-1), which acts as an oncogene. YAP1 is the effector of the Hippo pathway, which dysfunction can result in tumorigenesis and metastasis (Chan et al. 2014; Kovar et al. 2020). Moreover, both Wnt and Hh pathway components are significantly upregulated in metastatic cells compared to parental cell lines (Muff et al. 2015). Another study demonstrated the upregulation of target genes of both AKT/PI3K and Hh pathways in canine mammary osteosarcomas; this indicates that the interaction of these two pathways may be an important factor in osteosarcoma formation and proliferation (Pawlowski et al. 2011).

The Notch pathway has also been shown to influence the Hh pathway in the pathogenesis of osteosarcoma. DNMT3A, through methylation of miR-149, leads to its decreased activity, promoting overexpression of Notch components. This results in increased activity of the Hh pathway, which induces osteosarcoma's development and progression (Cheng and Wang 2022).

The role of the Hh pathway in the pathogenesis of osteosarcoma has also been shown in several studies assessing the preclinical efficacy of the Hh pathway inhibitors. Cyclopamine, a Smo inhibitor, promoted G1-phase arrest of the cell cycle, inhibited expression of cyclin D1, cyclin E1, SKP2, and pRb, and, as a result, restrained the growth of osteosarcoma in vitro (Hirotsu et al. 2010). In an animal model, it has been shown that cyclopamine decreased pulmonary osteosarcoma metastasis formation by 20% (Warzecha et al. 2012). Four acylguanidine and acylthiourea derivatives of cyclopamine, which have similar Smo-inhibiting properties, had been shown to have either cytotoxic or proliferation-inhibiting effects in osteosarcoma cell lines. The three drugs inhibiting proliferation decreased the expression of Gli1 and showed significant pro-apoptotic activity without causing severe side effects. Moreover, unlike the older generation of Smo inhibitors—cyclopamine, vismodegib, or sonidegib—they also effectively inhibit a chemoresistant form of Smo (Bernardini et al. 2018).

Examination of osteosarcoma cell viability revealed that treatment based on a combination of ATO and GANT61 (Gli inhibitors) or vismodegib (Smo inhibitor) decreased osteosarcoma cell migration. Moreover, inhibition of the osteosarcoma metastasis to the lung was observed during this combination treatment (Nagao-Kitamoto et al. 2015b). Decreased expressions of Gli1, Gli2, Ptch1, and PAX6 were observed after treatment of canine osteosarcoma cells using GANT61. Moreover, inhibition of cell growth was observed (Shahi et al. 2014). GANT61 has also been proven to inhibit the viability of certain osteosarcoma cell lines (Lo et al. 2014b) and to reduce the resistance of osteosarcoma cells to cisplatin in vivo (Chen et al. 2021). A Gli inhibitor ATO promotes apoptotic cell death in human osteosarcoma cells by accumulating DNA damage (Nakamura et al. 2013). Moreover, recent findings indicate that ATO inhibits the transcriptional activity of Gli2 and inhibits osteosarcoma cell invasion (Nagao-Kitamoto et al. 2015b).

Another study on patient-derived xenograft models of osteosarcoma evaluated the efficacy of treatment with saridegib, another Smo inhibitor. The drug effectively inhibited the canonical but not the non-canonical Hh pathway in the tumor and its microenvironment. The inhibition resulted in decreased expression of Ptch1 and Gli1, increased level of apoptosis, and decreased tumor weight and volume (Lo et al. 2014b).

Transcription of the genes associated with osteogenic differentiation is connected with the degree of the chromatin compaction (Montecino et al. 2020). There are different mechanisms that control chromatin organization in osteogenic cells, one of the major ones is a polycomb repressor complex 2 (PRC2) (Voigt et al. 2013; Shi et al. 2017).

PRC2 is involved in the modification of the chromatin during osteogenesis—it catalyzes the process of the trimethylation of histone H3 at lysine 27 (H3K27me3) and, as a result, induces chromatin condensation and transcriptional repression (Chamberlain et al. 2008). Enhancer of zeste homolog 2 (Ezh2) is one of the methylotransferases forming PRC2 (Shi et al. 2017). EZH2 is involved in the process of skeletal development, supporting self-renewal of mesenchymal stem cells and blocking osteogenic commitment of progenitor cells (Carrasco et al. 2023). It has been described that inactivation of Ezh2 leads to the activation of major osteogenic pathways and increased expression of bone formation-related genes and results in the pro-osteogenic effects (Dudakovic et al. 2020, 2015). A study revealed that the Ezh2 inhibitor-Tazometostat (EPZ6438), through the loss of H3K27me3 in the presence of osteogenic cues, enhanced osteogenic differentiation (Carrasco et al. 2023). Different results of Ezh2 inhibition have been described depending on the period of the Ezh2 inhibition (Carrasco et al. 2023). Short-term inactivation of Ezh2 (such as caused by Tazometostat-EPZ6438) activates the osteogenic process in the progenitor cells and stimulates bone formation in vivo, while persistent loss of Ezh may lead to a loss in the number of the osteoblasts (Dudakovic et al. 2020, 2016; Galvan et al. 2021).

### Ewing sarcoma

Ewing sarcoma (ES) is the second most frequent bone sarcoma affecting children and adolescents (Balamuth and Womer 2010; Grünewald et al. 2018). It occurs predominantly in bones (long bones, pelvis, chest wall, and spine) and to a much lower extent in soft tissues (W.C.o.T.E. Board 2020; Lézot et al. 2020). Ewing sarcoma is associated with chromosomal translocation—usually t(11;22) (q12;q24), which leads to *EWSR1–FLI1* genes fusion (Aurias et al. 1984). Nonetheless, in approximately 15–20% of Ewing sarcomas, *EWSR1* is fused with members of the ETS family other than FLI1, most frequently *ERG* (Sorensen et al. 1994).

Both survival and tumorigenesis of the Ewing sarcoma family of tumors are keyed to the function of *EWS-FLI1,* and it was shown that Gli1 is a transcriptional target of *EWS-FLI1* (Beauchamp et al. 2009). Data suggest that Gli1 upregulation by *EWS-FLI1* is Smo-independent, which indicates an involvement of the non-canonical activation pathway (Zwerner et al. 2008; Joo et al. 2009).

The efficacy of ATO, a direct Gli inhibitor, has been shown in both cell lines and xenograft models of the *EWS-FLI1* Ewing sarcoma. Its effects included cell cytotoxicity and inhibition of cell migration and invasion (Beauchamp et al. 2011) (Zhang et al. 2012). In vitro studies experiments demonstrated that another Gli inhibitor, GANT61, reduces the growth of Ewing sarcoma cells, mainly by inducing caspase-3/7-dependent cell apoptosis. The SK-N-LO cell line, characterized by the presence of the EWS-FLI1 fusion, was the most sensitive line to GANT61 treatment (Mullard et al. 2020).

### Chondrosarcoma

Chondrosarcoma is a malignant cartilage tumor, against which typically neither chemotherapy nor radiotherapy is effective (W.C.o.T.E. Board 2020; Fiorenza et al. 2002). Physiologically, Ihh and PTHrP (parathyroid hormone-related protein) regulate chondrocyte proliferation and differentiation. This signaling pathway is controlled through a negative feedback loop. A study showed that the Ihh–PTHrP pathway is dysregulated in chondrosarcoma cells, leading to constitutive ligand-dependent Hh signaling, as demonstrated by overexpression of Ptch1 and Gli1. However, no correlation was observed between the tumor grade and the level of Hh pathway components’ expression (Tiet et al. 2006). Another study demonstrated increased levels of Ihh mRNA compared to Shh mRNA and high levels of Ptch1, Smo, and Gli1 mRNA.

The efficacy of saridegib (Smo inhibitor) in the treatment of chondrosarcoma in primary xenografts was also evaluated. The observed effects included decreased volume and cellularity of the tumor, tumoral calcification, and decreased chondrocyte proliferation. The high efficacy of the treatment was likely due to the ligand-dependent nature of the Hh pathway present in chondrosarcoma (Campbell et al. 2014).

However, emerging evidence suggests that the non-canonical pathway activation might also play a role in its pathogenesis. It has been shown that Gli1 overexpression can be caused by the major vault protein (MVP) via mTOR/S6K1 signaling pathway (Wang et al. 2021). This might explain the unsatisfactory results of the clinical trials assessing the efficacy of Smo inhibitors—saridegib or vismodegib in patients with chondrosarcoma (Wagner et al. 2013; Italiano et al. 2013).

### Rhabdomyosarcoma 

Rhabdomyosarcoma is a high-grade tumor of skeletal myoblast-like cells and is the most common malignant soft-tissue sarcoma affecting children (W.C.o.T.E. Board 2020; Skapek et al. 2019; Dziuba et al. 2018). There are two major subtypes of RMS—embryonal rhabdomyosarcoma (ERMS) and alveolar rhabdomyosarcoma (ARMS) (Yechieli et al. 2021). ARMS samples' analysis demonstrated that most cases are connected with chromosomal translocation (Gallego Melcón and Sánchez de Toledo Codina 2007; Davis et al. 1994). Such chromosomal aberrations can lead to gene fusions, which are observed in ARMS, where in the majority of samples *PAX3/7–FOXO1* gene fusion is present (Kaleta et al. 2019). Chromosomal translocations have not been observed in ERMS cases; however, another study, which examined 12 embryonal rhabdomyosarcoma specimens from 10 patients, revealed gains and losses of some of the chromosomes or chromosomal regions. One of the most frequent ones was the loss of 9q22, a locus of *Ptch* (Bridge et al. 2000) (Roma et al. 2012). A cohort study revealed that in both ERMS and fusion-negative ARMS, *Ptch1*, *Gli1,* and *Gli3* genes have higher expression levels than in fusion-positive ARMS. This study also demonstrated a correlation between high expression of *Ptch1* and reduced overall survival in both ERMS and fusion-negative ARMS (Zibat et al. 2010). However, there is an ongoing discussion regarding whether the survival of ERMS patients is linked to *Gli1* and *Ptch1* expression level—microarray analysis of rhabdomyosarcoma samples showed that the survival rate, age, tumor stage, group, and the primary anatomic site did not correlate with the expression of *Gli1* mRNA transcripts (Pressey et al. 2011). Furthermore, this study did not identify any correlation between *Gli1* or *Ptch1* expression and poor clinical outcomes in ERMS and gene-fusion-negative ARMS patients (Pressey et al. 2011). However, the authors of this study emphasize the necessity of carrying out more trials on larger cohorts of patients to understand the molecular background of ERMS pathogenesis properly.

Regarding the efficacy of Hh inhibitors, an in vivo study was carried out to assess the effectiveness of sonidegib (Smo inhibitor) treatment in the embryonal subtype of rhabdomyosarcoma (ERMS) with a mutation in *Ptch*. Sonidegib had a significant antitumor effect in murine models of ERMS, as evidenced by a reduction of tumor growth in monotherapy and combined with pictilisib, a PI3K inhibitor. This effect of sonidegib treatment correlated with a decreased expression of Gli1 in vitro. Another study with vismodegib showed a similar effect (Geyer et al. 2018). Moreover, ATO, a Gli inhibitor, was identified to reduce viability and clonal growth and induce apoptosis of both embryonal and alveolar rhabdomyosarcoma cell lines (Boehme et al. 2016).

### Leiomyosarcoma

Leiomyosarcoma (LMS) is one of the most common soft-tissue sarcoma in adults, representing 10–20% of newly diagnosed cases. Uterine LMS is, in turn, the most common type of uterine sarcoma (George et al. 2018). Increased Smo, SuFu, and Gli1 expression was described in uterine LMS compared to normal myometrium (Garcia et al. 2016). Another study confirmed elevated levels of Smo and Gli1 and that the Hh pathway is deregulated in uterine LMS (Garcia et al. 2021). A study demonstrated that leiomyosarcoma cells showed decreased proliferation, migration, and invasion in response to treatment with Smo or Gli inhibitors (Garcia et al. 2020).

Interesting data come from analyses of different regulators of the Hh pathway in LMS. It has been shown that NKX6-1 plays an oncogenic role in LMS, and its overexpression modulates the Shh pathway, leading to the promotion of stem cell properties in tumor cells and poor prognosis. In vitro treatment of NKX6-1 overexpressing LMS cells with an inhibitor of the Shh pathway RU-SKI43 resulted in cell growth inhibition (Su et al. 2021). The effect of GANT61 (Gli inhibitor) on LMS was assessed using a leiomyosarcoma xenograft model. GANT61 caused significant regression of the leiomyosarcoma growth and decreased expression of Gli1 and its target genes: BMP4 and c-MYC (Garcia et al. 2022).

### Malignant rhabdoid tumor

Malignant rhabdoid tumors (MRT) are a group of mainly soft-tissue cancers which most commonly develop in the kidney or the brain but can be found in any body part. Those located in the brain are referred to as atypical teratoid/rhabdoid tumors (ATRT). MRT mainly affect infants and is believed to develop during embryogenesis. They remain one of the most lethal pediatric cancers and have a particularly bad prognosis in case of metastases (W.C.o.T.E. Board 2020; Custers et al. 2021).

Most ATRTs are characterized by a biallelic mutation of the *SMARCB1* gene and loss of encoded protein INI1/SNF5 (Frühwald et al. 2016). A recent meta-analysis has divided all MRT into three main subgroups based on their molecular and clinical features. One of them, ATRT-SHH, is characterized by an overexpression of Shh pathway components (such as Gli1 and Ptch1) and members of the Notch pathway (Ho et al. 2020). A study indicates that in MRTs, the Shh pathway is activated through the non-canonical pathway. The lack of SNF5 protein, typically involved in limiting Gli1 expression, leads to Gli1 overexpression and drives the growth of cancer cells. This theory was supported by the in vivo treatment of MRT with a small-molecule inhibitor of Gli, which led to the decrease of Gli1 levels and inhibition of tumor growth, whereas Smo inhibitors had no effect (Jagani et al. 2010) (see Table 1).

**Table 1:** Hedgehog pathway components expression and effect of pathway inhibition in vitro and in vivo in various sarcoma*s*

	Overexpression of Hh components in cell lines	Overexpression of Hh components in human tumors	Effect of Hh inhibition	Other pathways involved
Osteosarcoma	Shh Ihh Ptch1 Smo Gli	Shh Ihh Ptch1 Smo Gli	Cyclopamine (SMO inhibitor): Inhibition of growth and metastasis formation in preclinical models Vismodegib (SMO inhibitor): Decreased cell migration and metastasis formation (when used in combination treatment) Saridegib (SMO inhibitor): Effective inhibition of canonical pathway, noneffective inhibition of non-canonical pathway in xenograft model ATO (Gli inhibitor) Decreased cell migration and metastasis formation in preclinical models (when used in combination treatment) Increased apoptosis, reduced invasion GANT61 (Gli inhibitor) Reduction of osteosarcoma cells' resistance to cisplatin Inhibition of cell growth Inhibition of certain OS cell lines viability reduction of cell migration and metastasis formation in combination treatment in preclinical models Emodin Lowered radioresistance of osteosarcoma cells Degalactotigonin Suppression of osteosarcoma cells	Wnt pathway Notch pathway Hippo pathway MAPK pathway PI3K/AKT/mTOR pathway
Ewing sarcoma	Gli	Gli	GANT61 (Gli inhibitor) Reduced cell growth in preclinical models (particularly in those with the presence of the EWS-FLI1 Fusion gene) ATO (Gli inhibitor) Increased cytotoxicity, reduced cell migration and invasion in preclinical models of EWS-FLI1 Ewing-sarcoma	EWS–FLI1 pathway IGF–1R pathway
Chondrosarcoma	Ptch1 Gli1	Ptch1 Gli1	Saridegib (Smo inhibitor) Decreased volume and cellularity of the tumor Decreased tumoral calcification Decreased chondrocyte proliferation in xenograft models	Ihh–PTHrP pathway PI3K/AKT/mTOR pathway SRC pathway
Rhabdomyosarcoma	Ptch1 Gli1 Gli3	Ptch1 Gli1 Gli3	Vismodegib, Sonidegib (SMO inhibitors) Reduced tumor growth Reduced number of proliferating cells in ERMS with a mutation in Ptch in preclinical models ATO (Gli inhibitor) Increased cells apoptosis Reduced viability and clonal growth in preclinical models of both ERMS and ARMS	Wnt pathway Notch pathway Hippo pathway p53 pathway
Leiomyosarcoma	Smo Gli	Smo SuFu Gli1	**GANT61** (Gli inhibitor) Regression of leiomyosarcoma growth on xenograft model	
Malignant rhabdoid tumor (ATRT-SHH subtype)	Gli1	Gli1	small molecule inhibitor of Gli Inhibition of tumor growth in in vivo treatment Smo-inhibitors No effect on tumor growth inhibition	Notch pathway

## Shh pathway inhibitors in the treatment of sarcomas

### Smo inhibitors

Smo inhibitors include cyclopamine and its derivatives and analogues: vismodegib, saridegib (IPI-926), and sonidegib (erismodegib, LDE225). While cyclopamine is chemically unstable (Tremblay et al. 2009) and has significant side effects (Warzecha et al. 2012), the newer Smo inhibitors are characterized by a more favorable pharmacokinetic profile and, in some cases, a higher potency (Tremblay et al. 2009; Kumar and Fuchs 2015).

Cyclopamine inhibited growth and metastasis formation in preclinical models of osteosarcoma (Hirotsu et al. 2010; Warzecha et al. 2012). Vismodegib, used in combination treatment, was also successful in preclinical models, leading to a decrease in cell migration and metastasis formation in osteosarcoma (Nagao-Kitamoto et al. 2015b), and a reduction of tumor growth and the number of proliferating cells in the embryonal subtype of rhabdomyosarcoma with a mutation in *Ptch* (Geyer et al. 2018). Saridegib decreased tumor size and increased apoptosis in the preclinical models of osteosarcoma associated only with the canonical activation of the Hh pathway (Lo et al. 2014b) Sonidegib significantly reduced tumor growth and the number of proliferating cells in the preclinical models of the embryonal subtype of rhabdomyosarcoma with a mutation in *Ptch* (Geyer et al. 2018). Promising results have also been obtained in a study assessing the efficacy of four new cyclopamine derivatives in osteosarcoma cell lines. They significantly induced apoptosis and also effectively inhibited a chemoresistant form of Smo, unlike the older generations of Smo inhibitors (Bernardini et al. 2018).

While vismodegib and sonidegib have been approved by FDA as agents targeting the Hh pathway in the treatment of basal cell carcinoma, none of the Smo inhibitors has been approved for therapy of any sarcomas associated with Hh pathway dysfunction (Meiss et al. 2018; Casey et al. 2017) due to the lack of efficacy in clinical trials. The efficacy of vismodegib was assessed in a clinical trial conducted to determine if dual inhibition of the Notch and Hh pathways would result in a synergistic antitumor effect in advanced sarcomas (Gounder et al. 2022). Patients received a combination of vismodegib and a Notch inhibitor RO4929097 or Notch inhibitor alone. No patients had an objective response, and there was no difference in progression-free or overall survival between treatment arms. Paired tumor biopsies from a subset of patients demonstrated decreased expression of cleaved Notch and decreased phosphorylated Akt, suggesting successful inhibition of the gamma-secretase enzyme leading to downregulation of Notch signaling. Contrary, only two out of ten patients had a substantial decrease in *Gli1* expression, implying that inhibition of the canonical Hh pathway was not very effective (Gounder et al. 2022).

It is important to note that the non-canonical pathway activation happens independently of Smo. Its inhibitors have thus no effect on the pathway in cases where this method of activation is predominant. This might explain the unsatisfactory results of the clinical trials assessing the efficacy of saridegib and vismodegib in patients with chondrosarcoma, where a significant role of the non-canonical activation is suspected (Wang et al. 2021; Wagner et al. 2013; Italiano et al. 2013).

Another possible explanation for the lack of efficacy of Smo inhibitors is the presence of specific mutations in the target protein. Several solutions have been proposed to overcome this issue: development of second-generation Smo inhibitors that would retain their inhibitory effect despite the presence of the mutation, for example, by targeting a different domain of the protein; targeting downstream molecules of Smo, such as Gli transcription factors; and finally genetic prescreening before initiating Smo inhibitor therapy (Nguyen and Cho 2022).

Another promising strategy could be the simultaneous inhibition of both the upstream and downstream levels of the Shh pathway. A study showed that a synthetic isoflavone, which targets Smo and Gli1 at the same time, had a significant anti-tumor effect both in in vitro and in vivo models of medulloblastoma. This form of therapy also has the potential to decrease the toxicity of individual Shh pathway inhibitors and should therefore be examined in sarcoma models (Lospinoso Severini et al. 2019).

### Gli inhibitors

Gli inhibitors include GANT61 and arsenic trioxide (ATO). There is an ongoing discussion about their potential therapeutic value, particularly in the case of the non-canonical activation of the Shh pathway, where Smo inhibitor therapies have no effect (Beauchamp and Uren 2012).

GANT61 reduced cell growth in preclinical models of Ewing sarcoma, particularly the ones characterized by the EWS–FLI1 fusion gene (Mullard et al. 2020). It also reduced cell viability (Lo et al. 2014b; Shahi et al. 2014) as well as the resistance to cisplatin (Chen et al. 2021) in monotherapy and cell migration and metastasis formation in combination treatment (Nagao-Kitamoto et al. 2015b), in preclinical models of osteosarcoma. Finally, GANT61 caused a regression of growth in preclinical models of leiomyosarcoma (Garcia et al. 2022).

ATO in monotherapy in preclinical models of EWS–FLI1 Ewing sarcoma led to cell cytotoxicity, reduced both cell migration and invasion (Beauchamp et al. 2011; Zhang et al. 2012). Moreover, in preclinical osteosarcoma models, ATO has been observed to increase apoptosis and reduce invasion (Nagao-Kitamoto et al. 2015b; Nakamura et al. 2013). A study showed that targeting both embryonal and alveolar rhabdomyosarcoma with ATO resulted in increased apoptosis, reduced viability, and reduced clonal growth (Boehme et al. 2016). ATO combined with GANT61 or vismodegib reduced cell migration and metastasis formation in preclinical osteosarcoma models (Nagao-Kitamoto et al. 2015b).

These findings suggest that Gli inhibitors may have a potential therapeutic value in treating sarcomas associated with elevated Gli levels, particularly in the case of the non-canonical activation of the Shh pathway, where Smo inhibitors have no effect. However, while the FDA has approved ATO for the treatment of acute promyelocytic leukemia (Ferrara et al. 2022), neither drug has been registered as a treatment method for sarcomas. The significant cytotoxicity caused by Gli inhibitors might be a possible setback (Sigafoos et al. 2021).

### Other drugs

Emodin (1, 3, 8-trihydroxy-6-methylanthraquinone) is a natural anthraquinone derivative which has been reported to have various desired pharmacological effects, such as an anti-neoplastic, antioxidant, anti-inflammatory, and anti-apoptotic potential (Semwal et al. 2021). Regarding emodin’s anticancer activity, its effects include induction of apoptosis and cell cycle arrest, anti-metastasis activity, and reversion of multidrug resistance (Dong et al. 2016). A study showed that emodin, through inhibition of the Shh pathway, can partially reverse the radioresistance of osteosarcoma cells. In this study, two cell lines have been used: human osteosarcoma cell line MG63 and, produced through 30-repeat low-dose X-ray irradiation cycles, cell line MG63R (radioresistant OS cells). The study revealed that emodin treatment before irradiation inhibited the nuclear translocation of Gli1 in MG63 cells and lowered the levels of Shh and BCL2 in MG63R cells (Qu et al. 2017). BCL2 is an antiapoptotic protein whose function has been linked to the mitochondrial pathway and has been described to be expressed at high levels in osteosarcoma cells (Chen et al. 2015). It has been described that BCL2 protein protects osteosarcoma cells from apoptosis, and on this account, silencing BCL-2 may have a positive outcome on the effectiveness of therapeutic strategies in osteosarcoma (Zhao et al. 2009).

Degalactotigonin (DGT), a substance extracted from the plant *Solanum nigrum* L., has been shown to inhibit the Hh pathway through GSK3 beta inactivation. This leads to the suppression of osteosarcoma proliferation and metastasis (Zhao et al. 2018). Glycogen synthase kinase-3β (GSK3β) is a serine/threonine protein kinase which levels have been described to have a direct impact on osteosarcoma cells (Tang et al. 2012). A study on patient-derived xenograft models has shown that inhibition of GSK-3β leads to inhibition of the NF- κB pathway and results in the apoptosis of osteosarcoma cells (Tang et al. 2012).

## Conclusions and discussion

Dysregulation of the Hh pathway, leading to overexpression of its components, has been observed in some sarcomas, including osteosarcoma, Ewing-sarcoma, chondrosarcoma, rhabdomyosarcoma, leiomyosarcoma, and malignant rhabdoid tumor. This points to the critical role played by the Hh pathway in the tumorigenesis of sarcomas; however, Hh components do not seem to be the core mechanism in the pathogenesis of these tumors. Subsequently, the efficacy of sarcomas treatment with Hh inhibitors was assessed. Both in vitro and in vivo studies showed promising results, but they have not yet been confirmed in clinical trials. We believe that the observed lack of effect may result from two factors: the non-canonical pathway activation and interactions with other signaling pathways.

Due to its mechanism, the non-canonical pathway activation is less sensitive to Smo inhibition, making Gli inhibitors the only potentially effective Hh-targeted treatment in this group of sarcomas. Understanding which mechanism of Hh pathway activation is present in each sarcoma subtype (Table 2) is crucial to properly plan and execute preclinical experiments and clinical studies with Hh-targeted agents. Interactions between the Shh pathway and pathways, such as Wnt and Notch, create a complex network where activation of one pathway can increase the expression of the others’ components. Several connection points between Hh and Notch, Wnt, or TGF-β pathways show a reciprocal synergism contributing to tumorigenesis in various tumors but have not yet been thoroughly studied in sarcoma (Pelullo et al. 2019). This is thought to be one of the mechanisms of target-specific treatment evasion (Roma et al. 2012).

Another aspect of the Shh pathway’s role in the treatment of sarcomas is its possible influence on susceptibility to immunotherapy. Reduced expression of the Hedgehog signaling pathway and the presence of CD8 + T cells have been shown to correlate with the best clinical outcome in patients with sarcoma undergoing immunotherapy (D’Angelo et al. 2022). Similar observations were also made in other tumors, such as gastric, breast, and basal cell carcinoma. It has been shown that the inhibition of Hh signaling results in decreased PD-L1 expression and tumor cell proliferation in mouse-derived gastric cancer organoids (Chakrabarti et al. 2018). Shh pathway inhibitors used in basal cell carcinoma have led to tumor regression accompanied by beneficial changes in the tumour’s microenvironment, such as upregulation of MHC class I expression, alteration of the local cytokine network, and infiltration of CD8 + T cells (Otsuka et al. 2015). Changes in the tumor microenvironment connected with Hh pathway inhibition have also been investigated in immunocompetent breast cancer murine models. It has been shown that Hh inhibition resulted in the reduction of immune-suppressive cells and an increased number of cytotoxic immune cells (Hanna et al. 2019). These effects could be potentially beneficial for treating sarcomas, which are generally considered “cold” tumors with a low number of infiltrating lymphocytes and rather immunosuppressive microenvironment (Petitprez et al. 2020).

A better understanding of the role played by the Shh pathway in the pathogenesis of sarcomas is necessary. We propose that further research should primarily focus on the role of the non-canonical pathway and the development of its inhibitors as well as potential combination therapies, which would simultaneously target not only the Shh pathway but also other signaling pathways. Such studies could be used to develop an effective therapy for sarcomas connected with Shh pathway dysregulations and thus help solve one of oncology’s most prominent problems (see Table 2).

**Table 2:** Proposed mechanism of Hedgehog pathway activation in various types of sarcomas

Type of sarcoma	Proposed hedgehog pathway activation (canonical, non-canonical, both)	Explanation
Osteosarcoma	Both	Increased Smo expression and susceptibility to Smo inhibition Other cell lines: lack of susceptibility to Smo inhibition but susceptibility to direct Gli inhibition
Ewing sarcoma	Non-canonical	Gli1 upregulation by the EWS–FLI1 fusion gene, independent of Smo
Chondrosarcoma	Both	Dysregulation of the Ihh–PTHrP pathway leads to constitutive ligand-dependent Hh signaling Gli1 overexpression due to MVP activity via mTOR/S6K1 signaling
Rhabdomyosarcoma (ERMS and fusion gene-negative ARMS)	Canonical	Mutation in Ptch1, leading to increased Ptch1, Gli1 and Gli3 expression Susceptibility to Smo inhibition
Leiomyosarcoma	Canonical	Increased Smo expression Susceptibility to Smo inhibition
Malignant rhabdoid tumor (ATRT-SHH subtype)	Non-canonical	Lower expression of the SNF5 protein leads to Gli1 overexpression

## Data Availability

Not applicable.
